# Reducing Nonradiative Recombination Losses in Tin-Based Perovskite LEDs Utilizing a Self-Assembled Monolayer

**DOI:** 10.1021/acsami.5c15797

**Published:** 2025-10-22

**Authors:** Sergio Galve-Lahoz, Jesús Sánchez-Diaz, Ece Aktas, Jhonatan Rodriguez-Pereira, Antonio Abate, Juan Luis Delgado, Iván Mora-Seró

**Affiliations:** † Institute of Advanced Materials (INAM), 16748 University Jaume I, Av. Vicent Sos Baynat, S/N, Castellón de la Plana 12071, Spain; ‡ Polymat, University of the Basque Country UPV/EHU, Donostia-San Sebastian 20018, Spain; § Department of Chemical, Materials and Production Engineering, 28340 University of Naples Federico II, Piazzale Via Tecchio 80, Fuorigrotta, Napoli 80125, Italy; ∥ Center of Materials and Nanotechnologies, Faculty of Chemical Technology, 48252 University of Pardubice, nám. Cs. legií 565, Pardubice 53002, Czech Republic; ⊥ Central European Institute of Technology, Brno University of Technology, Purkynova 123, Brno 612 00, Czech Republic; ○ Ikerbasque, Basque Foundation for Science, Bilbao 48013, Spain

**Keywords:** tin halide perovskite, lead-free LEDs, SAM, interface engineering, TEA_2_SnI_4_, PEDOT:PSS, hole selective layer

## Abstract

Tin-based perovskites are emerging as less-toxic alternatives to their lead-based counterparts for optoelectronic devices, such as solar cells and light-emitting diodes (LEDs). However, despite their great potential, the efficiency of pure red tin-based perovskite LEDs (Sn-LEDs) still lags behind that of lead-based perovskite LEDs (Pb-LEDs), partly due to the poor electron blocking at the PEDOT:PSS/perovskite interface. This leads to detrimental nonradiative recombination pathways that limit the performance of the LEDs. In this study, we replaced the conventional PEDOT:PSS layer with the self-assembled monolayer (SAM) EADR03, presenting, to the best of our knowledge, the first report of a SAM employed as a hole-selective layer in Sn-LEDs. EADR03 simultaneously acted as an efficient electron-blocking and hole-injecting layer, thereby reducing interfacial recombination losses and enhancing the LEDs’ performance. As a result, we achieved a 3-fold enhancement in external quantum efficiency, propelling the advancement of more efficient tin-based perovskite LEDs.

## Graphical abstract

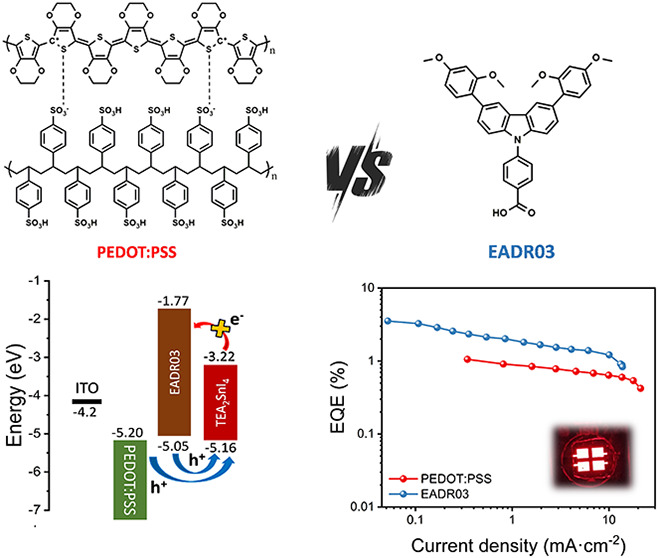

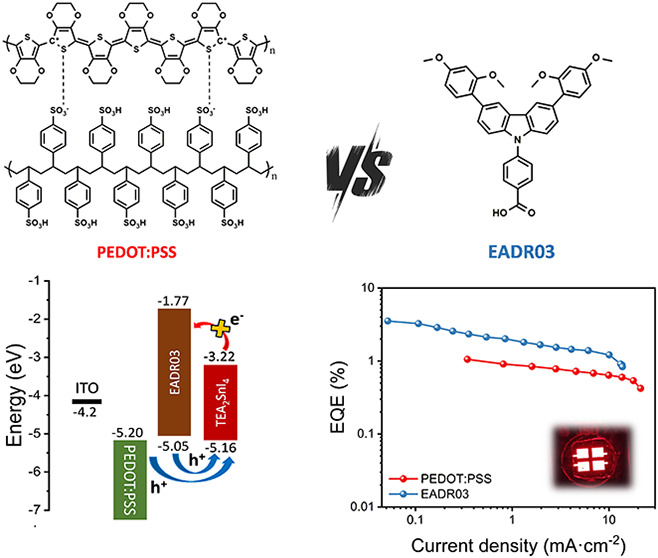

## Introduction

Metal halide perovskites have emerged as promising semiconductor materials due to their remarkable potential in different optoelectronic applications, including solar cells (SCs) and light-emitting diodes (LEDs).^1,2^ Among them, tin-based halide perovskites (Sn-HPs) have attracted growing interest as a less toxic alternative to their lead-based counterparts while still offering excellent optoelectronic properties.^3,4^ Although Sn-based solar cells have shown significant progress during the last years, the potential of Sn-based perovskite LEDs (Sn-LEDs) still remains largely unexplored.^5^ Their development is mainly hindered by the typical challenges presented by Sn-HPs, such as the easy oxidation of Sn^2+^ to Sn^4+^, and their fast crystallization rate that leads to defect-rich structures.^6,7^ Accordingly, most reports have been focusing on finding suitable additives and reducing agents that can regulate the crystallization rate and inhibit the oxidation process, respectively.^8–11^ However, a less-mentioned issue related to Sn-LEDs concerns the hole-selective layer (HSL). So far, poly­(3,4-ethylenedioxythiophene) polystyrenesulfonate (PEDOT:PSS) has been the only HSL reported in Sn-LEDs, mainly due to its high conductivity and good wettability, among other advantages. Nevertheless, it presents a series of limitations, such as high parasitic absorption,^12^ hygroscopic behavior, and strong acidity that can damage ITO electrodes and compromise the long-term stability of the devices.^13,14^ Furthermore, PEDOT:PSS is a strongly p-doped polymer with high conductivity and semimetallic behavior.^15^ Consequently, its interface with perovskite typically induces nonradiative exciton quenching via energy transfer and/or trap-assisted charge recombination, thus reducing the performance of the devices.^16–18^

Despite these issues, only a few reports have addressed this problematic interface in Sn-LEDs so far.^19,20^ A common approach widely explored in Pb-based perovskite LEDs (Pb-LEDs) relies on the incorporation of electron-blocking interlayers that prevent the direct contact between PEDOT:PSS and perovskite, effectively reducing the electron leakage.^17,21–23^ However, such interlayers rarely overcome the other intrinsic limitations of the PEDOT:PSS. A more promising strategy involves the full replacement of PEDOT:PSS with alternative HSLs. In this context, self-assembled monolayers (SAMs) have shown improved optoelectronic properties in Pb-LEDs,^24–27^ but they have not yet been reported in Sn-LEDs, likely due to the wettability challenges associated with SAMs and to the shallow valence band maximum (VBM) of Sn-HPs.^28^ In any case, suppressing interfacial recombination while ensuring high-quality perovskite films is essential to achieving significant improvements in the performance of Sn-LEDs. Taking this into account, we were then motivated to find an HSL that can effectively address both challenges.

In this work, we replaced PEDOT:PSS with SAM 4-(3,6-bis­(2,4-dimethoxyphenyl)-9H-carbazol-9-yl)­benzoic acid (EADR03), using it as the sole HSL in Sn-LEDs. The good wettability of EADR03 enables the formation of smooth Sn-HP films, while its proper energy alignment effectively suppresses electron transfer at the interface, thereby reducing nonradiative recombination. As a result, a 3-fold enhancement in the maximum external quantum efficiency (EQE) of the devices, ranging from 1% to 3.5%, was obtained. This is, to the best of our knowledge, the first report of SAM-based Sn-LEDs, paving the way for a future search for novel interfacial layers or alternative HSL materials that can boost the efficiency and scalability of Sn-LEDs.

## Experimental Section

### Materials

All of the reagents used in the photovoltaic study were obtained from commercial suppliers in high purity and employed without further purification: 2-Thiopheneethylammonium iodide (TEAI, 99.99%) was purchased from Greatcell Solar Materials. Tin­(II) iodide (SnI_2_, 99.99%), *N*,*N*-dimethylformamide (DMF, 99.8%), and dimethyl sulfoxide (DMSO, 99.8%) were purchased from Sigma-Aldrich. PEDOT:PSS Al 4083 aqueous solution was purchased from Heraeus. 4-(3,6-Bis­(2,4-dimethoxyphenyl)-9H-carbazol-9-yl)­benzoic acid (EADR03, 99%) and 2,4,6-Tris­[3-(diphenylphosphinyl)­phenyl]- 1,3,5-triazine (PO-T2T, 99%) were purchased from Lumtec. Aluminum pellets (Al, 99.99%) were purchased from Lesker. Prepatterned ITO glass substrates (20 × 20 × 1 mm, 20 Ohm·sq^–1^) were purchased from Visiontek.

### Device Fabrication

2.0 × 2.0 cm prepatterned ITO substrates were cleaned in subsequent ultrasonic baths, 15 min each. First, the substrates were washed with water and soap and then rinsed with milli-Q water. Afterward, they were cleaned with acetone and isopropanol, and finally dried with N_2_ flow. A 25-min UV-Ozone treatment was performed strictly before the hole selective layer (HSL) deposition. For HSL deposition, a PEDOT:PSS solution was filtered with a 0.45 μm PVDF filter and spin-coated on top of the ITO substrates at 3500 rpm·s^–1^ for 40 s, followed by annealing at 125 °C for 30 min. The SAM layer was formed by statically spin-coating 100 μL of 4-(3,6-bis­(2,4-dimethoxyphenyl)-9H-carbazol-9-yl)­benzoic acid (EADR03) dissolved in DMF at a concentration of 1 mM (with a 5-s waiting time, 4000 rpm for 30 s). This was followed by annealing at 150 °C for 15 min. The substrates were then immediately introduced into a N_2_-filled glovebox for the perovskite layer deposition. TEA_2_SnI_4_ perovskite precursor solution was prepared at 0.16 M using stoichiometric amounts of TEAI and SnI_2_ dissolved in a DMSO:DMF (1:9 V/V) mixed solvent and stirred overnight. Sn powder was added to the solution at a concentration of 5 mg/mL. The perovskite solution was filtered and spin-coated on top of PEDOT:PSS at 4000 rpm for 60 s. No antisolvent was used, and the film was prepared by a two-step annealing at 60 °C for 1 min and 90 °C for 12 min. Finally, 40 nm of PO-T2T and 110 nm of Al were deposited by thermal evaporation.

## Results and Discussion

The chemical structures of PEDOT:PSS and EADR03 are presented in Figure 1a. Figure 1b represents the energy levels of PEDOT:PSS, EADR03, and the 2D perovskite used in this work, 2-thiophenethylammonium tin-iodide (TEA_2_SnI_4_). All of the values are summarized in Table S1. Compared to other SAMs, EADR03 presents favorable energy levels to facilitate efficient hole injection into the Sn-based perovskite layer while simultaneously preventing the electron leakage from the perovskite conduction band minimum (CBM), thus promoting favorable radiative recombination.^28^

**Figure 1:**
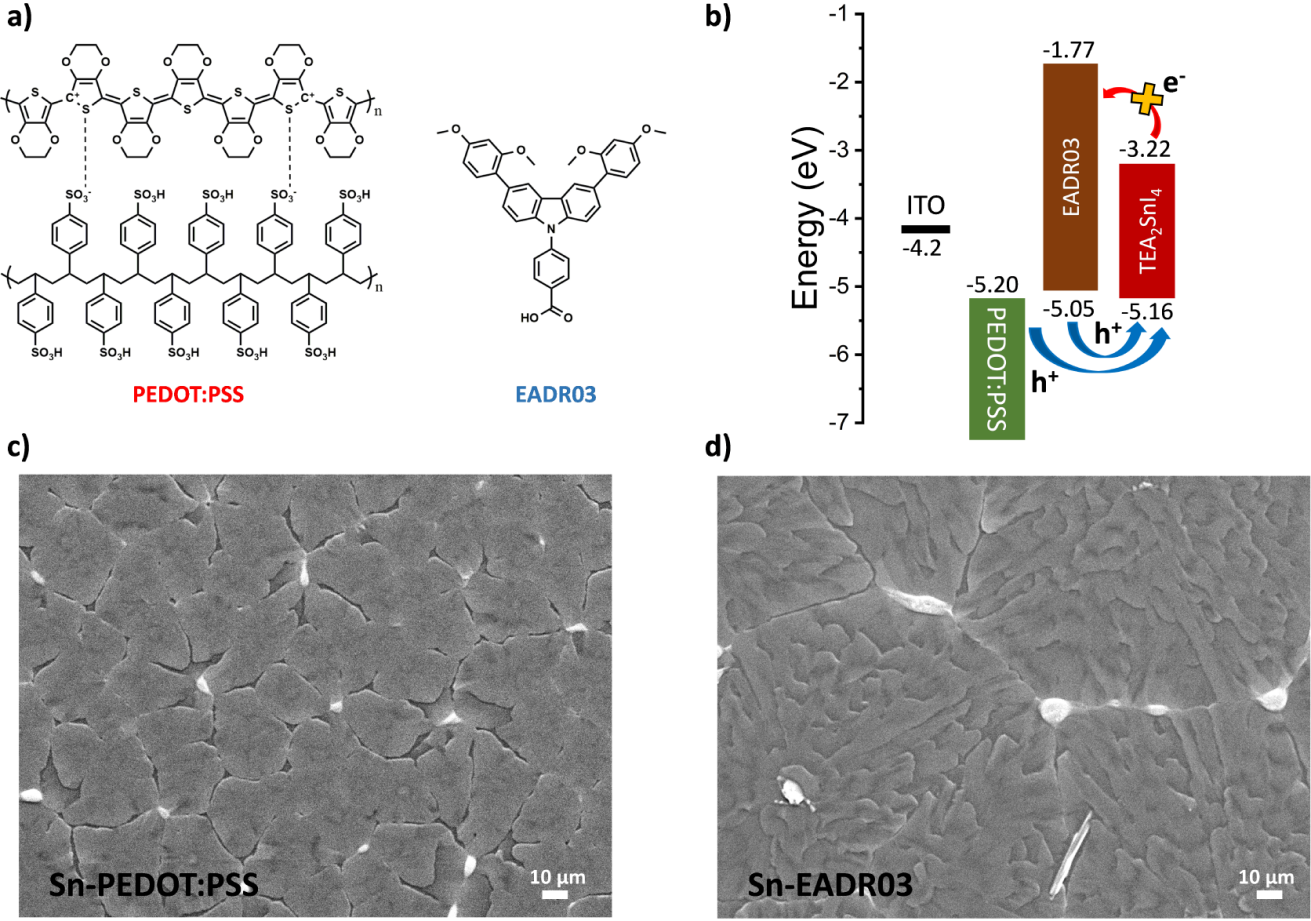
(a) Chemical structures of PEDOT:PSS and EADR03. (b) Energy band diagram for ITO, PEDOT:PSS, EADR03, and TEA_2_SnI_4_, values extracted from refs. 9, 29 , and^30^. Top-view SEM images of TEA_2_SnI_4_ on top of (c) PEDOT:PSS and (d) EADR03.

Due to its chemical structure, EADR03 can be chemically attached to the ITO substrate through an ester-type covalent bonding between its carboxylic acid moiety and the hydroxyl groups present on the ITO surface.^30,31^ This chemical attachment ensures a stable interface, modifying the surface properties for a proper perovskite deposition without compromising the charge transfer at the interface.^32^ X-ray photoelectron spectroscopy (XPS) measurements were performed to verify the surface chemical composition. Figure S1 shows the high-resolution (HR) spectrum of C 1s for the ITO/EADR03 surface. The C 1s HR spectrum was curve-fitted into five components corresponding to C–(C,H) at 284.80 eV, C–N–C at 286.10 eV, C–O–C at 286.80 eV, HO—C=O at 288.38 eV, and O=C—O at 289.95 eV. The peaks attributed to C–N–C and HO—C=O confirm the formation of the EADR03 film, and the presence of the O=C—O peak verifies the formation of a covalent ester bond between the carboxylic group of EADR03 and the hydroxyl groups present on the ITO surface. Additionally, the higher In^3+^/In–OH ratio in the ITO/EADR03 film (2.6%) compared to the bare ITO film (2.0%) indicates fewer free hydroxyl groups on the ITO surface, further confirming the formation of the chemical bond (see Figure S2). Note that although the chemical bonding of EADR03 to the ITO hydroxyl groups is demonstrated, the resulting layer should not be strictly referred to as a monolayer, as it is deposited by spin coating. Figure S3 shows the contact angles of water droplets on the ITO (88.2°) and ITO/EADR03 (54.6°) surfaces. The increased hydrophilicity upon the surface modification by EADR03 can improve perovskite’s wettability, addressing one of the main challenges associated with SAM-type molecules in Sn-based systems, where poor wettability typically results in poor film coverage and the formation of low-quality perovskite films.^33^ From hereafter, and to avoid confusion, TEA_2_SnI_4_ films deposited on PEDOT:PSS and EADR03 will be referred to as Sn-PEDOT:PSS and Sn-EADR03, respectively. Note that all films were deposited without using an antisolvent step, making the procedure more compatible with future upscaling approaches, as previously described by our group.^10^

To evaluate the perovskite morphologies on the different layers, we performed scanning electron microscopy (SEM) measurements. Figure 1c,d shows the top-view SEM images of Sn-PEDOT:PSS and Sn-EADR03 films, and lower magnification images with grain size histograms are also provided in Figure S4. The Sn-PEDOT:PSS film presented a poor and nonhomogeneous morphology, with incomplete film coverage. In contrast, Sn-EADR03 displayed a better film quality, with fewer pinholes and a more compact and uniform grain arrangement. This improved morphology can be attributed to effective interactions between the perovskite precursors and the EADR03 layer during the film formation, leading to a better crystallization process.

In order to confirm the crystallinity of the perovskite, X-ray diffraction (XRD) analysis was performed and is presented in Figure S5. Both Sn-PEDOT:PSS and Sn-EADR03 films exhibited the same 00*l* (*l* = 2, 4, 6, 8, 10, and 12) diffraction peaks, confirming the formation of the 2D crystal structure,^34^ and no significant change in the preferential crystalline orientation is observed.

To further assess the chemical quality of the films, we performed XPS measurements on both the Sn-PEDOT:PSS and Sn-EADR03 films. As shown in Figure S6 and Tables S2 and S3, perovskite films deposited on EADR03 exhibited a slightly lower amount of Sn^4+^ compared to those grown on PEDOT:PSS (31.5% vs 34%, respectively).^26^ This difference may suggest that EADR03 contributes to the formation of a less defective film, as the presence of Sn^4+^ is typically associated with the oxidation of Sn^2+^ and the formation of intrinsic defects in Sn-HPs. Additionally, the I^–^/I_2_ ratio was evaluated, and it was found to be higher for Sn-EADR03 films (26.8) compared to Sn-PEDOT:PSS films (10.5), pointing to a better chemical stability of the former. A lower I^–^/I_2_ ratio reflects a higher presence of molecular I_2_, which is a well-known byproduct resultant from the Sn-HPs degradation process,^35^ suggesting also a premature degradation. We also calculated the overall I:Sn atomic ratio, which, according to the TEA_2_SnI_4_ stoichiometry, should be 4:1. However, it was found to be 3.02 for Sn-PEDOT:PSS and 3.33 for Sn-EADR03. These discrepancies may result from iodide losses during film formation or from partial degradation during sample shipment for characterization. In any case, the XPS analysis suggests that replacing PEDOT:PSS with EADR03 improves the chemical quality of the final perovskite layer, as reflected by the higher I^–^/I_2_ ratio and the I:Sn stoichiometry closer to the theoretical value.

To understand the effect of the different HSLs on the optical properties and carrier dynamics of the perovskite films, we conducted ultraviolet–visible absorption (UV–vis), steady-state photoluminescence quantum yield (PLQY), and time-resolved PL spectroscopy (TRPL) measurements. Both Sn-PEDOT:PSS and Sn-EADR03 films showed similar narrow excitonic absorption peaks located at 618 nm (see Figure S7) and similar normalized PL spectra (see Figure S8), with photoluminescence emission peaks centered at 630 nm, but Sn-EADR03 presents higher steady-state PL intensity (Figure 2a). The PLQY of Sn-EADR03 (3.4%) was significantly higher compared to Sn-PEDOT:PSS (1.5%) (see Figure 2a). This discrepancy can be partly attributed to the highly p-doped nature of PEDOT:PSS. Du et al.^18^ showed that hole accumulation at the PEDOT:PSS/perovskite interface facilitates electron transfer from the perovskite’s CBM to the PEDOT:PSS layer, inducing nonradiative exciton quenching. In contrast, EADR03 can suppress this quenching pathway while simultaneously improving the perovskite film morphology, as shown in Figure 1c,d. Furthermore, Aranda et al. recently reported that the chemical interaction between the OH– groups present at the ITO substrate and the carboxylic groups of EADR03 prevents the accumulation of positively charged ions/vacancies at the interface, reducing the interfacial recombination.^36^ Therefore, we attribute the enhanced PLQY to a more favorable interface with suppressed ionic accumulation and fewer nonradiative recombination channels.^37–39^ Moreover, the TRPL spectra shown in Figure 2b support this. The shorter PL decay observed for Sn-PEDOT:PSS suggests undesirable electron transfer processes from perovskite’s CBM to PEDOT:PSS. In contrast, EADR03 suppresses this nonradiative recombination channel, leading to longer carrier lifetimes and favoring radiative recombination within the perovskite layer.^40,41^ The longer PL decay of Sn-EADR03 can also be associated with a lower density of structural defects, which act as nonradiative recombination centers,^42^ consistent with the improved perovskite morphology and inferring enhanced optoelectronic properties for Sn-EADR03.

**Figure 2:**
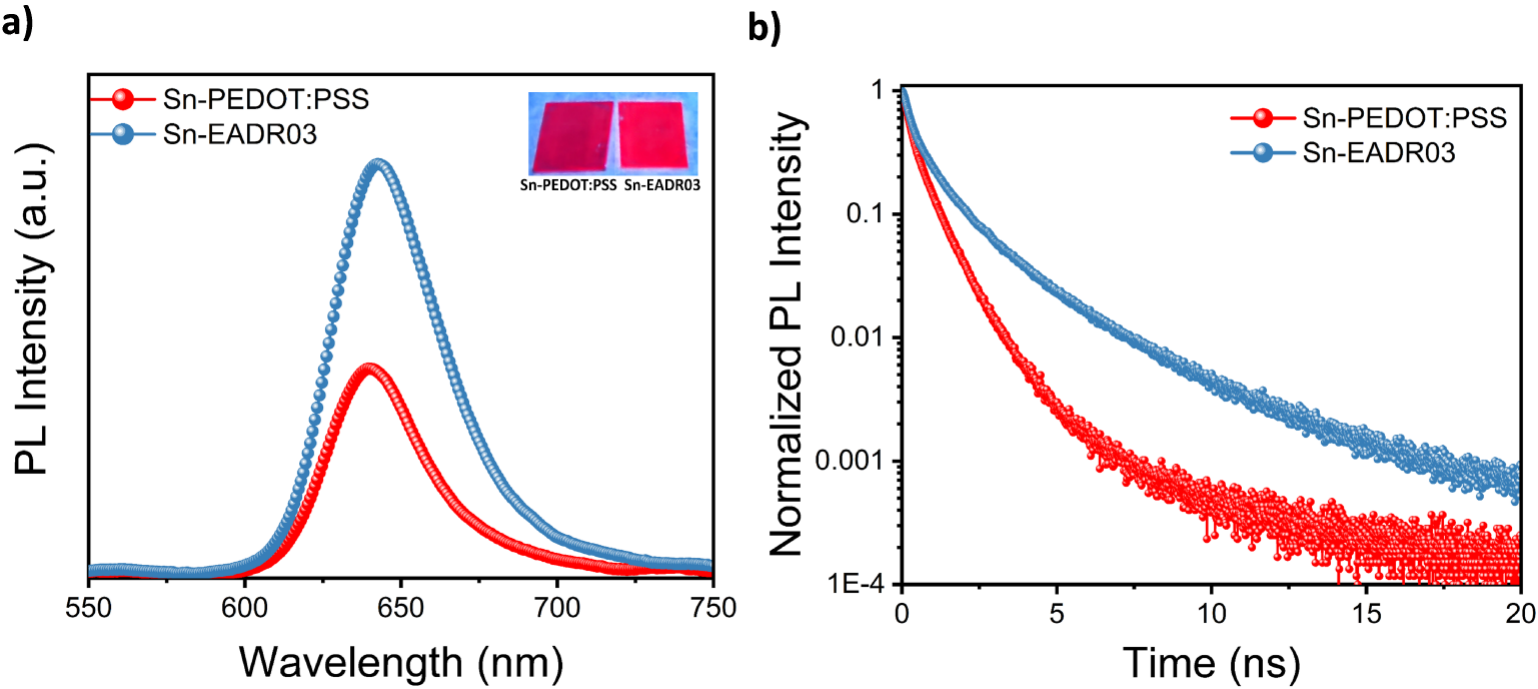
Optical properties of TEA_2_SnI_4_ films deposited on PEDOT:PSS or EADR03. (a) PLQY spectra and (b) TRPL spectra.

Furthermore, Sn-based LEDs were fabricated with a p-i-n configuration: ITO/HSL/TEA_2_SnI_4_/PO-T2T/Al, as shown in Figure 3a. Both control and EADR03-based devices exhibited electroluminescence (EL) peaks centered at 636 nm with LED color coordinates of (0.710, 0.290), matching the pure red vertex defined by the Commission Internationale de l’Eclairage (CIE) *REC*. 2020 standards (see Figure 3b,c).

**Figure 3:**
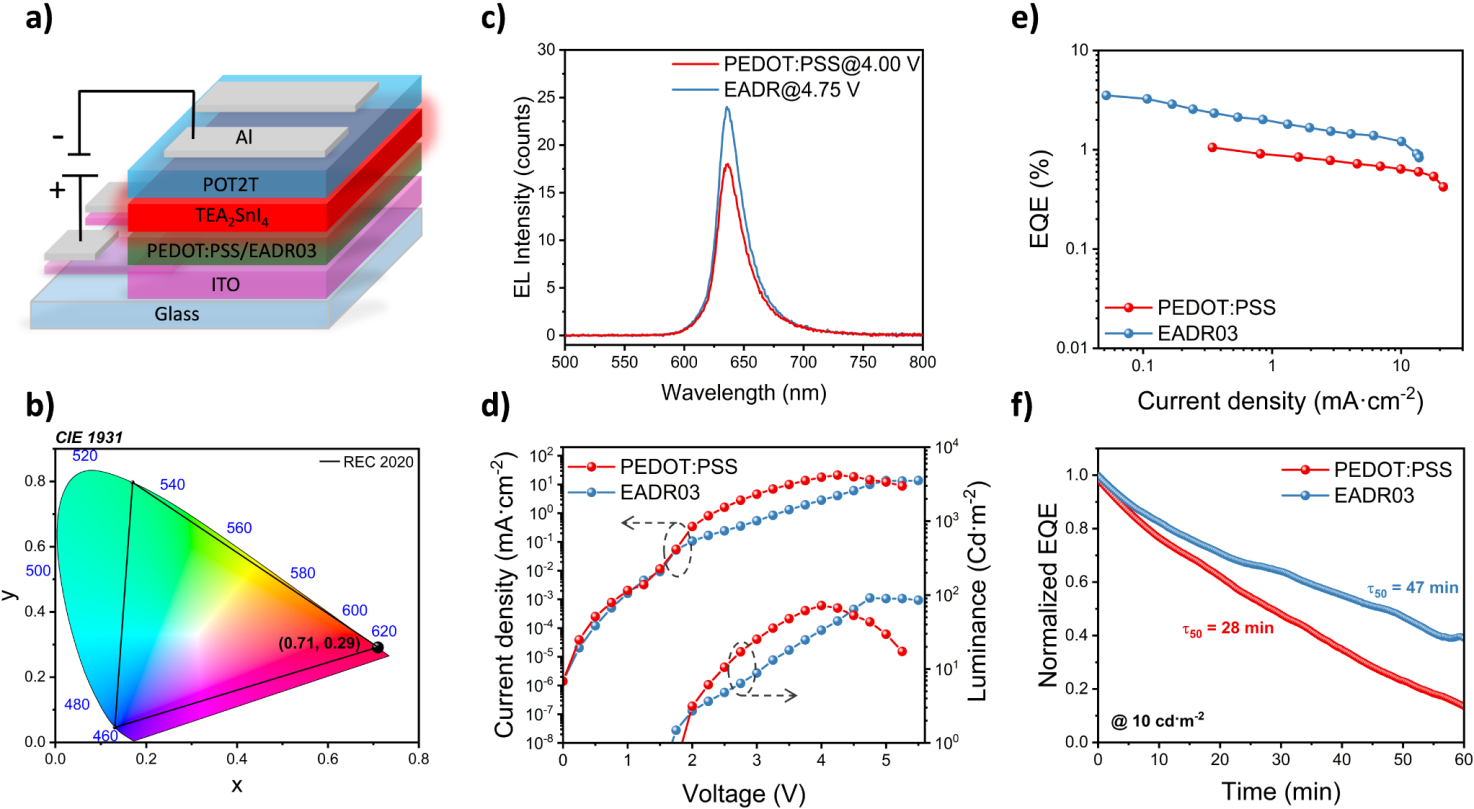
(a) Sn-LED device architecture. (b) CIE 1931 color chromaticity diagram of the Sn-LEDs. (c–f) Properties of the Sn-LEDs using PEDOT:PSS or EADR03 as HSL, namely (c) electroluminescence spectra, (d) current density–voltage-luminance curves, (e) EQE vs current density, and (f) normalized EQE-operational time at continuous working conditions, keeping constant the injected current that provides an initial luminance of 10 cd·m^–2^.

Moreover, although Sn-EADR03 presents lower luminance than Sn-PEDOT:PSS over a range of applied voltages, the luminance is obtained with a significantly lower amount of injected current, implying improved charge injection efficiency (see Figure 3d). As a consequence, and as shown in Figure 3e, Sn-PEDOT:PSS exhibited a maximum EQE of 1%, while Sn-EADR03 achieved a 3-fold enhancement in EQE, reaching up to 3.5%. In addition, it is worth highlighting that at high applied voltages, Sn-EADR03 reached a higher maximum luminance of 90 cd·m^–2^, compared to the 70 cd·m^–2^ obtained by Sn-PEDOT:PSS (see Figure 3d).

Figure 3e presents the results for the champion devices, and a statistical distribution of the maximum EQEs is also shown in Figure S8. Notably, EADR03-based devices retained EQE values above 1% even at high current densities as high as 10 mA·cm^–2^, thereby decreasing the high EQE roll-off typically observed in some of the state-of-the-art Sn-LEDs under high current operation.^9,43^ Moreover, as has been noted, Sn-PEDOT:PSS exhibited higher average current densities at the same applied voltages than Sn-EADR03. This behavior can be ascribed to the high conductivity of the PEDOT:PSS layer combined with current leakages caused by ineffective electron blocking at the PEDOT:PSS/TEA_2_SnI_4_ interface. These nonradiative leakage pathways not only limit the EQE but also increase the current density required to achieve the desired luminance, increasing thermal stress and potentially compromising long-term device stability. To verify this consideration, the operational stability of the encapsulated devices in ambient air (25 °C and 60% RH) at a constant injected current density that provides an initial brightness of 10 cd·m^–2^ was studied (see Figure 3f). Control devices presented a half-life stability (T_50_) of 28 min, whereas EADR03-based devices demonstrated superior stability, with a T_50_ of 47 min. Here, T_50_ is defined as the time under continuous operation at which the initial EQE drops to 50%. The reduced stability of control devices may be attributed to both the high hygroscopicity of PEDOT:PSS and its limited electron-blocking ability, which enables nonradiative leakage pathways. On the one hand, the high hygroscopicity can promote the adsorption of water molecules and accelerate the degradation processes of perovskite, and on the other hand, the increased nonradiative recombination processes may intensify the thermal stress of the devices and compromise their long-term stability.

## Conclusions

In summary, we present the first report of Sn-LEDs using a SAM (EADR03) as HSL. The perovskite films deposited on EADR03 showed improved morphology compared to those deposited on PEDOT:PSS, and, more importantly, the unfavorable PEDOT:PSS/TEA_2_SnI_4_ interface was resolved. EADR03 not only enables efficient hole injection but also effectively suppresses electron leakage, thus promoting radiative recombination and improving the device performance. This work demonstrates the potential of SAM as HSLs for Sn-LEDs and represents a step toward more efficient and stable Pb-free halide perovskite LEDs.

## Supplementary Material



## Notes

XPS and water contact angles of ITO and ITO/EADR03 films; SEM, XRD, XPS and UV–vis absorption of perovskite films; distribution of peak EQEs of LEDs (PDF)

## ∇

Department of Physics, University of Oxford, Clarendon Laboratory, Parks Road, Oxford OX1 3PU, UK
