# Supplementary material for: Reducing Nonradiative Recombination Losses in Tin-Based Perovskite LEDs Utilizing a Self-Assembled Monolayer
Source: ACS Appl Mater Interfaces. 2025 Oct 22;17(44):60937–43. doi: 10.1021/acsami.5c15797 (PMC12598692; doi:10.1021/acsami.5c15797)
Supplement: Supplementary file 1 [file am5c15797_si_001.pdf]

Supporting Information

Reducing Non-Radiative Recombination Losses in Tin-Based Perovskite LEDs Utilizing a Self-Assembled Monolayer

Sergio Galve-Lahoz,[1,2,#] Jesús Sánchez-Diaz,[1,#] Ece Aktas,[3,†] Jhonatan Rodriguez-Pereira,[4,5] Antonio Abate,[3] Juan Luis Delgado[2,6] and Iván Mora-Seró[1*]*

[1] Institute of Advanced Materials (INAM), University Jaume I, Av. Vicent Sos Baynat, s/n, 12071, Castellón de la Plana, Spain

[2] Polymat, University of the Basque Country UPV/EHU, 20018 Donostia-San Sebastian, Spain

[3] Department of Chemical, Materials and Production Engineering, University of Naples Federico II, Piazzale Via Tecchio 80, 80125 Fuorigrotta, Italy

[4] Center of Materials and Nanotechnologies, Faculty of Chemical Technology, University of Pardubice, nám. Cs. legií 565, Pardubice, 53002, Czech Republic

[5] Central European Institute of Technology, Brno University of Technology, Purkynova 123, Brno, 612 00, Czech Republic

[6] Ikerbasque, Basque Foundation for Science, Bilbao 48013, Spain

[†] Present address: Department of Physics, University of Oxford, Clarendon Laboratory, Parks Road, Oxford OX1 3PU, UK

# Both authors contributed equally to this work.

*Corresponding authors: sero@uji.es, juanluis.delgado@polymat.eu

Structural characterization

X-ray diffraction (XRD)

XRD patterns of the films were collected using a powder X-ray diffractometer (D8 Advance, Bruker-AXS) in a Bragg-Bretano geometry. Cu K_{α} X-ray radiation was employed (λ_1 = 1.5406 Å, λ_2 = 1.5444 Å, I_2/I_1 = 0.5), with a tube voltage and intensity of 40 kV and 40 mA, respectively. The goniometer arm length was 217.5 mm, and a divergence slit of 0.6° was used. A BRUKER-binary V3 detector was employed to scan from 5.0° to 70.0° (2θ °), with a scan step size of 0.02 (2θ °) and a counting time of 75 s per step. All measurements were performed at room temperature (298 K).

Scanning Electron Microscope (SEM)

The topographical images were taken using a field emission scanning electron microscope (FEG-SEM) JEOL 3100F operated at 15 kV. The SEM images were recorded from films deposited on top of ITO covered substrates.

X-ray Photoelectron Spectroscopy (XPS)

The surface chemical composition and electronic state of the different films were determined by XPS (ESCA-2SR, Scienta-Omicron). Spectra were recorded using monochromatic Al Kα = 1486.6 eV operated at 200 W. The charge was controlled with the charge neutralizer (CN-10) operated at 5 A and 1 eV. The binding energy scale was referenced to adventitious carbon (284.8 eV). CasaXPS processing software (Casa software Ltd) was used to analyze the data, and the quantitative analysis was made using sensitivity factors provided by the manufacturer.

Water contact angle

The water contact angle was measured using a Kruss DSA25E Drop Shape Analyzer by dropping a droplet of deionized water onto the different substrates. A 30-seconds video was recorded

registering the behavior of the drop until its stabilization and the contact angle of the stable drop was measured using the DSA25E built-in software.

Optoelectronic characterization

UV-Vis Absorption Spectra

The absorption spectra were recorded using a Varian Cary 300 UV/Vis absorption spectrophotometer. UV-Vis spectra were recorded from films deposited on glass substrates.

Steady-state Photoluminescence (PL) and Time-Resolved PL (TRPL)

The PL and TRPL characterization of the different fabricated samples was carried out at room temperature and ambient conditions. The different films were measured with an absolute PL quantum yield spectrometer Hamamatsu C9920-02.

Optoelectrical Characterization

The LEDs performance was measured with an external quantum efficiency (EQE) measurement system (Hamamatsu C9920-12).

Table S1. Work function (WF) and energy levels of the HOMO/VBM and LUMO/CBM for the studied materials. Values extracted from references [1-3].

Material	Φ (eV)	HOMO/VBM	LUMO/CBM	E_g (eV)
**ITO**	4.20	-	-	-
**ITO/EADR03**	3.75	-5.05	-1.77	3.28
**PEDOT:PSS**	5.20	-	-	-
**TEA_2SnI_4**	-	-5.16	-3.22	-1.94

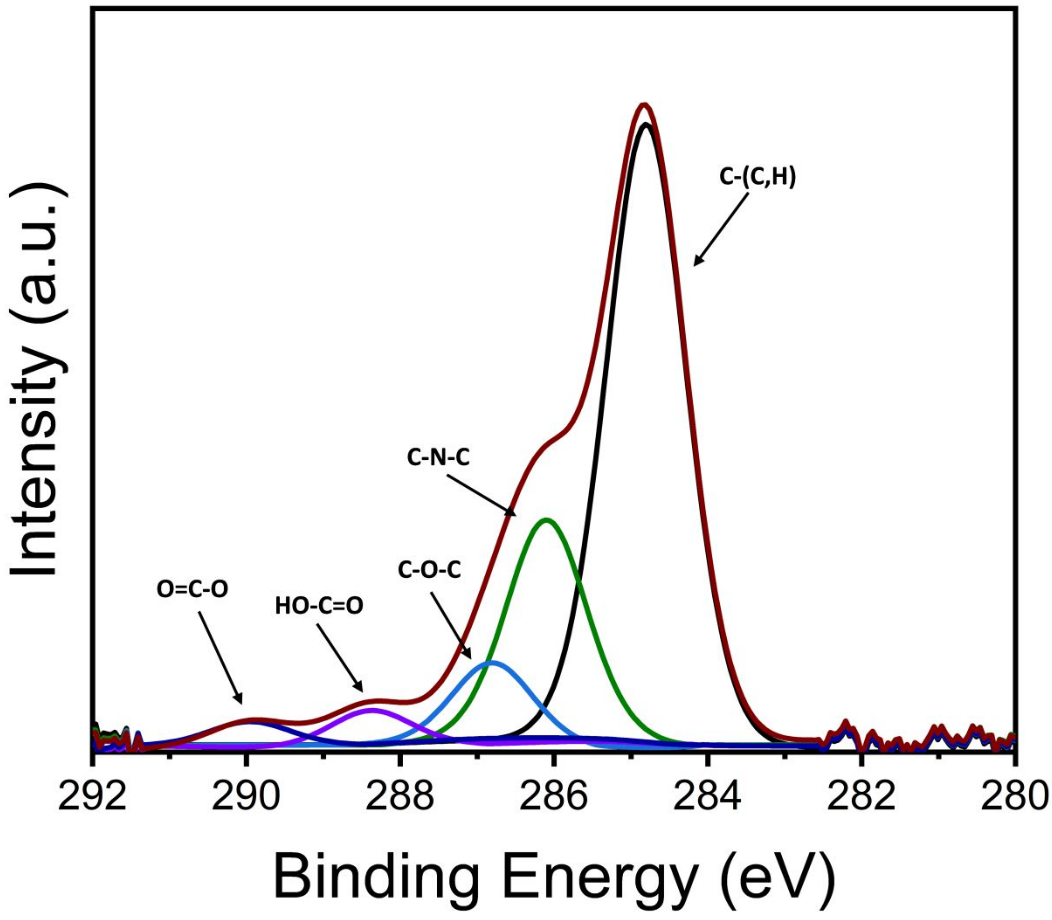

Figure S1. High-resolution XPS spectra of C 1s for ITO/EADR03.

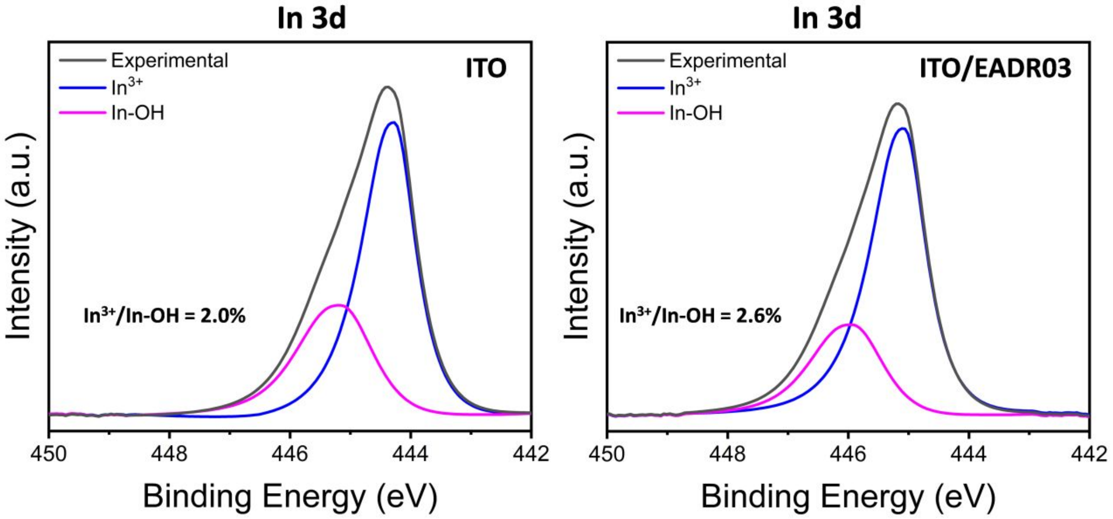

Figure S2. High-resolution XPS spectra of In 3d for ITO and ITO/EADR03 surfaces. The higher In^{3+}/In-OH ratio, along with the chemical shift towards higher binding energy confirms the chemical attachment of EADR03 to the ITO surface.

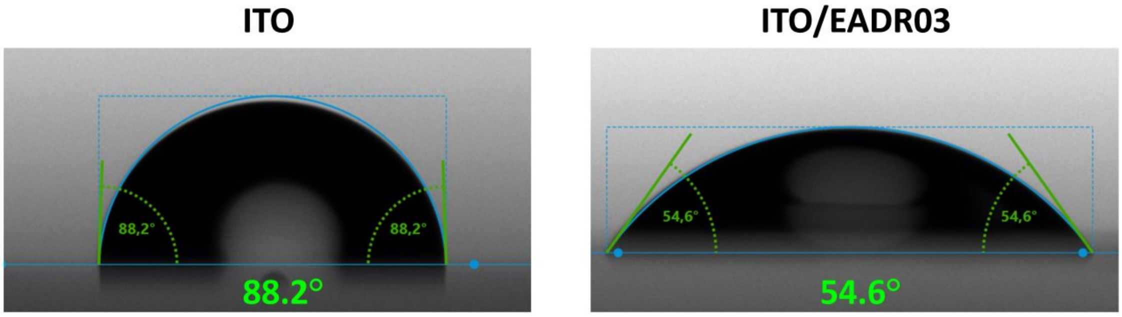

Figure S3. Contact angle measurements of water droplets on ITO and ITO/EADR03 surfaces.

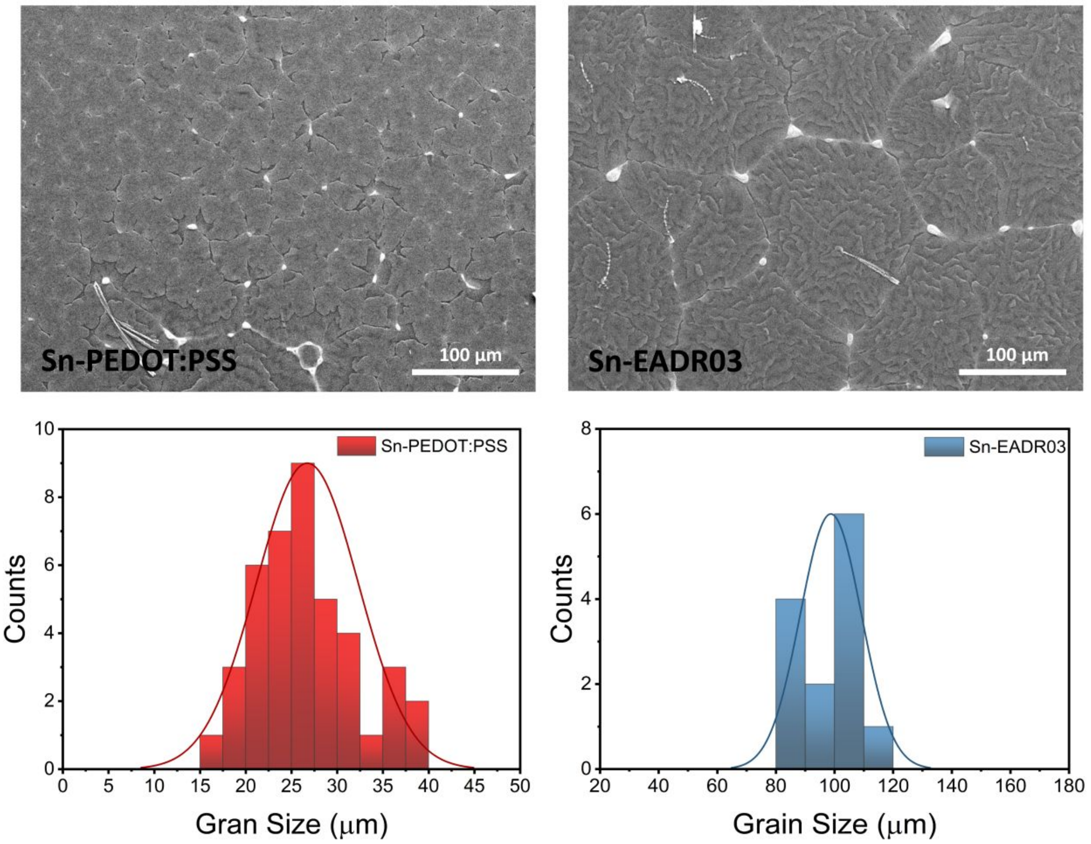

Figure S4. Low magnification top-view SEM images with their corresponding grain size distribution histograms. The average grain size for Sn-PEDOT:PSS is 27 ± 6 μm (21 % RSD) and in Sn-EADR03 it is 99 ± 10 μm (11 % RSD).

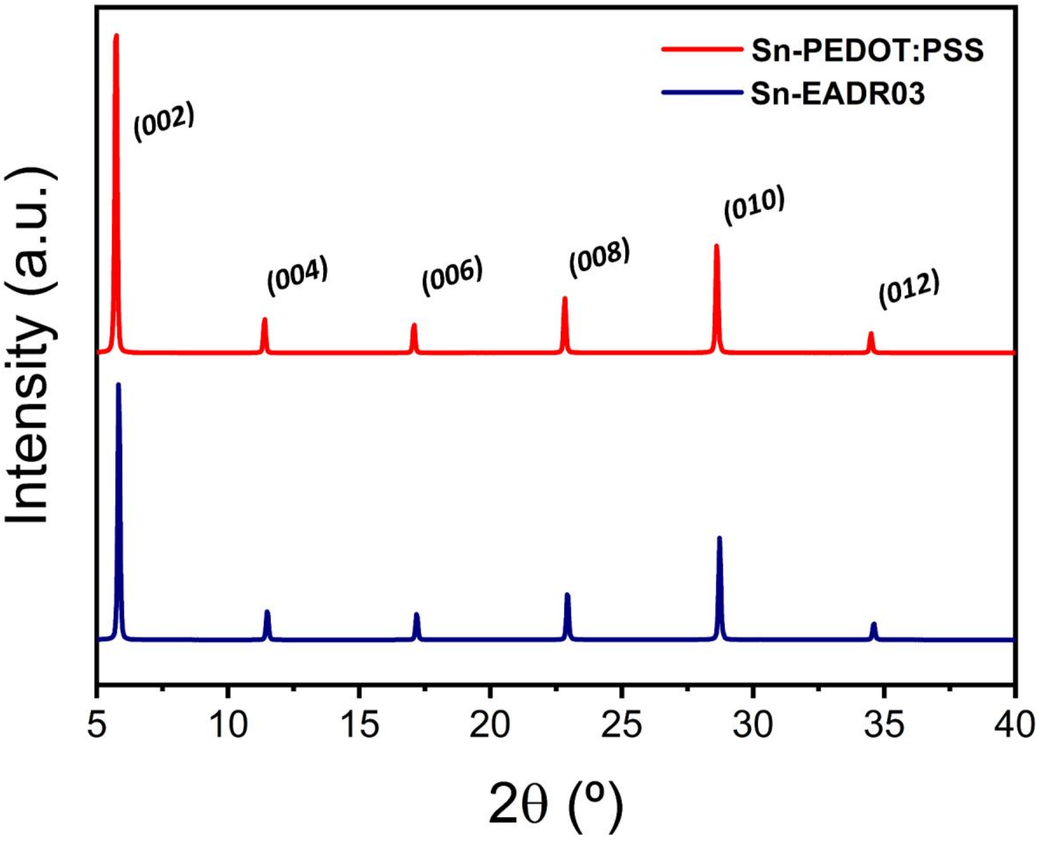

Figure S5. XRD patterns of Sn-PEDOT:PSS and Sn-EADR03.

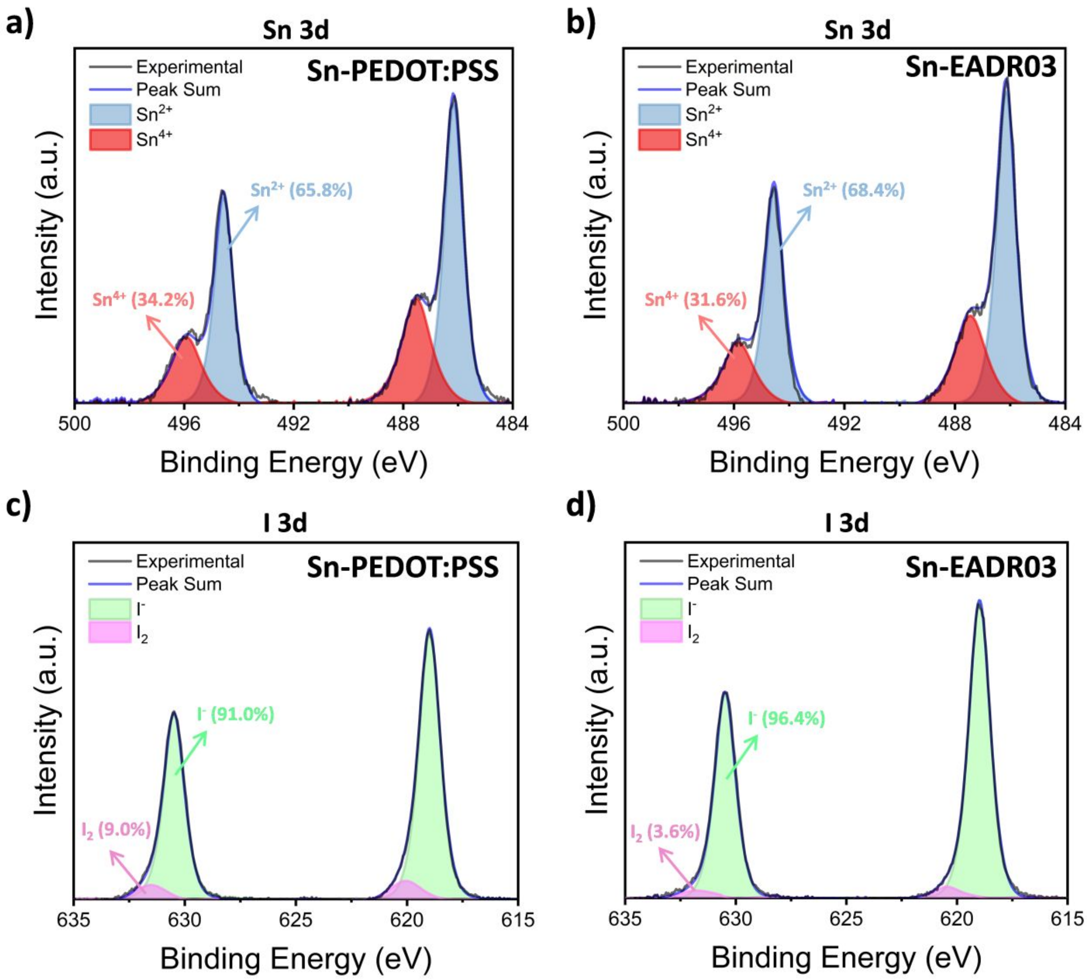

Figure S6. High-resolution XPS spectra of Sn 3d and I 3d for a, c) Sn-EADR03 and b, d) Sn-PEDOT:PSS films.

Table S2. Atomic concentration for different chemical species in Sn-PEDOT:PSS and Sn-EADR03 films. Extracted from XPS measurements.

	Atomic concentration (%)						
**Sample**	**Others (C, O, N, S)**	**Sn**	**I**	**Sn^{2+}**	**Sn^{4+}**	**I^-**	**I_2**
**Sn-PEDOT:PSS**	80.26	4.91	14.83	3.22	1.67	13.53	1.33
**Sn-EADR03**	79.16	4.81	16.03	3.29	1.52	15.43	0.58

Table S3. Atomic ratio for different chemical species in Sn-PEDOT:PSS and Sn-EADR03 films. Extracted from XPS measurements.

	Atomic ratio					
**Sample**	**Sn^{2+}/Sn^{4+}**	**I^-/I_2**	**$Sn^{2+}/(Sn^{2+}+Sn^{4+})$**	**$Sn^{4+}/(Sn^{2+}+Sn^{4+})$**	**$I^-/(I^-+I_2)$**	**$I_2/(I^-+I_2)$**
**Sn-PEDOT:PSS**	1.92	10.49	65.8%	34.2%	91.0%	9.0%
**Sn-EADR03**	2.16	26.78	68.4%	31.6%	96.4%	3.6%

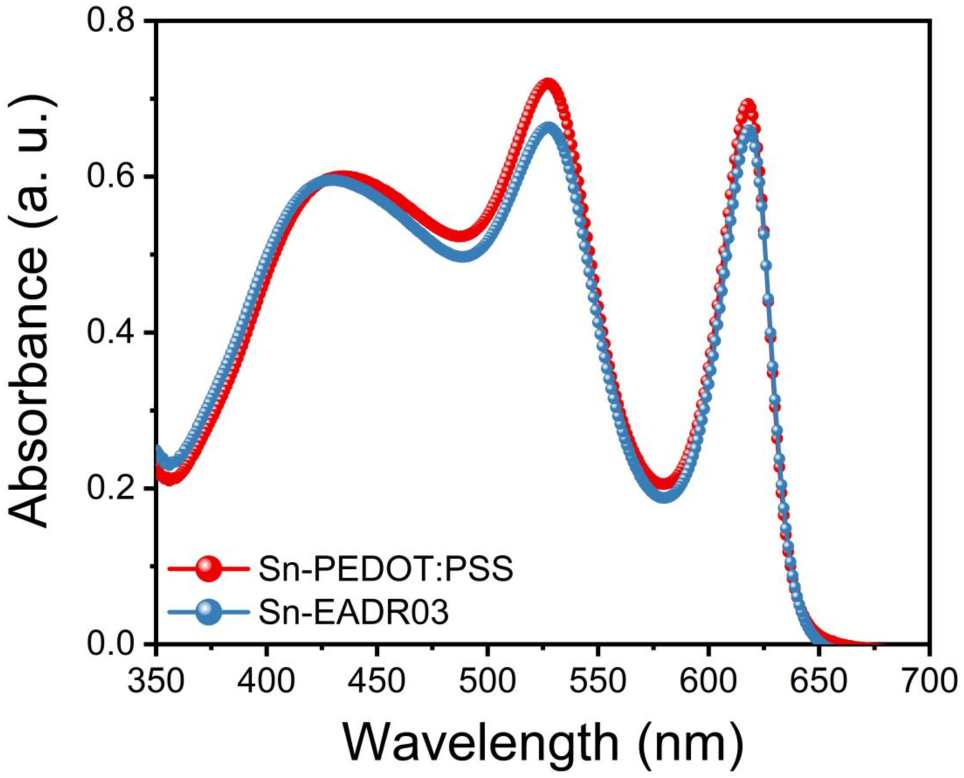

Figure S7. UV-vis absorption spectra of Sn-PEDOT:PSS and Sn-EADR03.

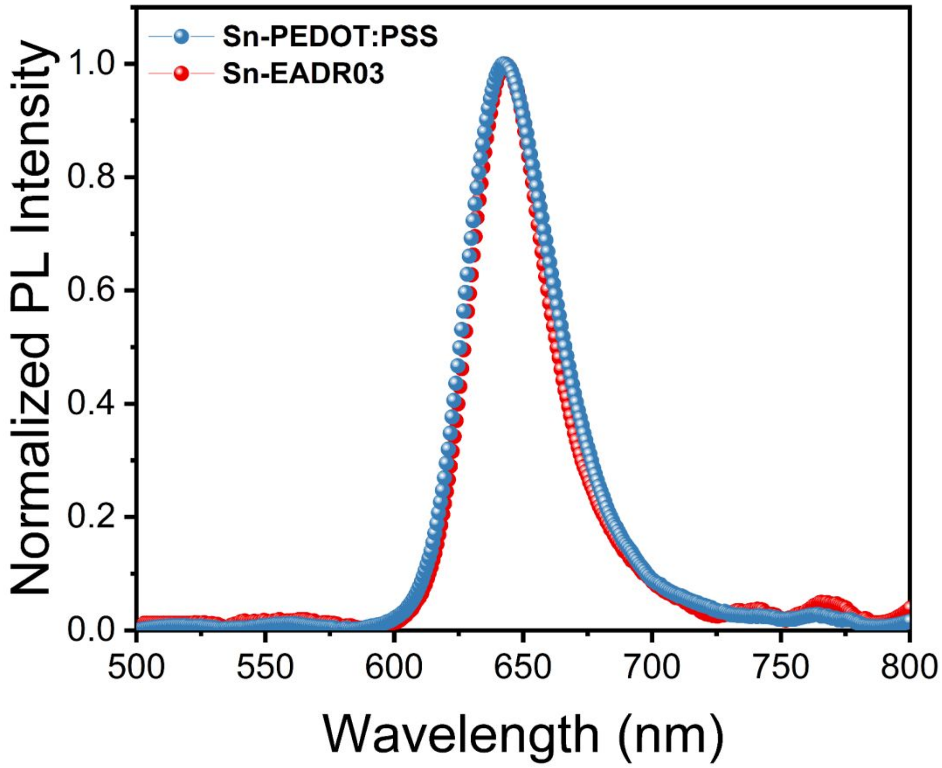

Figure S8: Normalized PL of **Figure 2a** in the main text.

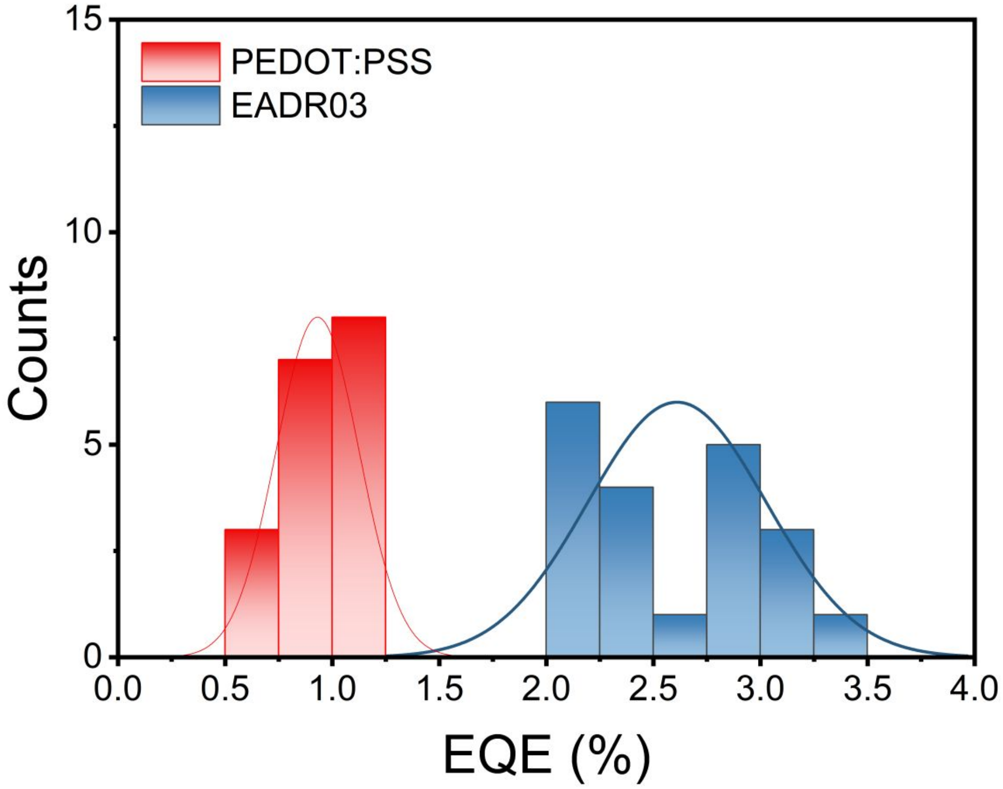

Figure S8. Histograms of peak EQEs.

1. Aktas, E.; Phung, N.; Köbler, H.; González, D. A.; Méndez, M.; Kafedjiska, I.; Turren-Cruz, S.-H.; Wenisch, R.; Lauermann, I.; Abate, A.; Palomares, E., Understanding the perovskite/self-assembled selective contact interface for ultra-stable and highly efficient p–i–n perovskite solar cells. *Energy & Environmental Science* **2021,** *14* (7), 3976-3985.
2. Dong, H.; Pang, S.; Zhang, Y.; Chen, D.; Zhu, W.; Xi, H.; Chang, J.; Zhang, J.; Zhang, C.; Hao, Y. Improving Electron Extraction Ability and Device Stability of Perovskite Solar Cells Using a Compatible PCBM/AZO Electron Transporting Bilayer *Nanomaterials* [Online], 2018.
3. Han, D.; Wang, J.; Agosta, L.; Zang, Z.; Zhao, B.; Kong, L.; Lu, H.; Mosquera-Lois, I.; Carnevali, V.; Dong, J.; Zhou, J.; Ji, H.; Pfeifer, L.; Zakeeruddin, S. M.; Yang, Y.; Wu, B.; Rothlisberger, U.; Yang, X.; Grätzel, M.; Wang, N., Tautomeric mixture coordination enables efficient lead-free perovskite LEDs. *Nature* **2023,** *622* (7983), 493-498.
